# Survey of Polymer Self-Healing Mechanisms in Perovskite Solar Cells

**DOI:** 10.3390/polym18010069

**Published:** 2025-12-26

**Authors:** Hayeon Lee, Zachary Lewis, Lars Christensen, Jianbo Gao, Dawen Li

**Affiliations:** 1 Department of Chemical and Biological Engineering, The University of Alabama, Tuscaloosa, AL 35487, USA; hlee110@crimson.ua.edu; 2 Department of Electrical and Computer Engineering, The University of Alabama, Tuscaloosa, AL 35487, USA; zblewis1@crimson.ua.edu (Z.L.); lzchristensen@crimson.ua.edu (L.C.); 3 Department of Chemistry, Yousef Haj-Ahmad Department of Engineering, Brock University, St. Catharines, ON L2S 3A1, Canada; jgao@brocku.ca

**Keywords:** perovskite solar cells, self-healing polymers, flexible and rigid substrates, mechanical stability, environmental durability, reversible and dynamic bonding

## Abstract

Perovskite solar cells (PSCs) have emerged as a rising next-generational photovoltaic technology due to low fabrication costs through solution processing as compared to traditional silicon solar cells and high-power conversion efficiency. However, the poor long-term operational stability due to environmental and mechanical degradation remains a hindrance to commercialization. Herein, self-healing polymer additives are utilized by researchers to enhance the photovoltaic performance of PSCs by enabling self-restorative behavior from physical damage or chemical degradation. This review explores the design and application of self-healing polymers in both flexible and rigid PSCs, contrasting the two main reversible bonding mechanisms: physical bonds, such as hydrogen bonds, and chemical bonds, such as dynamic covalent disulfide bonds. Physical bonds provide passive healing at ambient conditions; meanwhile, chemical bonds offer a stronger restoration under external stimuli such as heat or light. These polymers are exceptionally effective at mitigating mechanical stress and cracks in flexible PSCs and combating moisture-induced degradation in rigid PSCs. The applications of self-healing polymers are categorized based on substrate type, healing mechanism, and perovskite composition, with the benefits and limitations of each approach highlighted. Additionally, the review explores the potential of multifunctional self-healing polymers to passivate defects at the grain boundaries and on surface of perovskite films, thereby enhancing the overall photovoltaic performance.

## 1. Introduction

Perovskite solar cells (PSCs) are a leading next-generation photovoltaic technology due to their cost-effective manufacturing process, tunable electronic properties, and high-power conversion efficiencies (PCEs) that are comparable to their silicon counterparts. The perovskite family of materials is categorized by the chemical formula ABX_3_, with A being a large cation such as methylammonium (MA), formamidinium (FA) or a small cation of cesium, B being a central metal ion such as lead (Pb) or Tin (Sn), and X being a halogen ion such a bromine, chlorine, or iodine elements. Perovskites take the role of silicon, typically used in solar cells as the semiconductor light-absorbing material responsible for sunlight harvesting [1]. By replacing the silicon in the traditional solar cells with a perovskite film in a heterojunction structure, the costs associated with manufacturing these devices are significantly reduced, as they can be rapidly produced with more cost-efficient manufacturing techniques, such as high-speed coating and roll-to-roll printing [2]. Additionally, these thin-film solar cells can be fabricated on a rigid or flexible substrate, expanding the application of these devices from traditional solar panels to wearable flexible electronics [3]. However, both types of PSCs suffer from limitations that hinder commercialization, particularly long-term stability and durability [4]. Perovskite-based materials degrade in oxygen, under humidity, and at high temperatures, which often leads to a rapid loss of efficiency over time [5]. Since PSCs deteriorate in common atmospheric conditions, their applications are greatly limited. Water molecules can bond with lead ions or some cations to form new complexes that are incompatible with the perovskite formation, disassembling the lattice structure [6]. In order to address these stability issues, traditional methods to mitigate these degradation problems include interface engineering [7], surface passivation [8], device encapsulation [9], and compositional engineering [10]. While these approaches can improve the efficiency and stability of resulting devices, they are incapable of recovering the perovskite lattice and cell performance once degradation occurs.

Notably, perovskite materials without any additives exhibit intrinsic self-healing behavior under certain conditions, such as defect passivation during dark storage. This unique regenerative ability from the ionic lattice reformation and dynamic restoration of chemical bonds of perovskites invite us to rethink how we approach the stability of PSCs. Some PSCs have displayed performance improvements over time in specific environments known as “self-enhancement,” which is characterized by enhanced crystallinity, improved transport of charged carriers, and healing of trap states [11]. These findings suggest that self-repair is not a new concept for perovskites. Building on this insight, it is promising to explore extrinsic methods to support the innate tendencies of perovskite to regenerate from degradation.

In particular, the usage of self-healing polymer additives offers a promising pathway. Self-healing materials sufficiently repair damage, which can be utilized in both flexible and rigid PSCs to recover from environmental degradation and mechanical bending to improve operational long-term stability. By taking advantage of physical bonds that reform naturally and chemical bonds that require minimal energy input, these polymers can alleviate and often take advantage of originally unideal environmental conditions. Additionally, these polymer additives can be designed, synthesized, and processed to interact favorably with the perovskite absorber. As a result, incorporating self-healing polymers into PSCs is a promising method to overcome the short lifespan drawbacks of both rigid and flexible PSCs to advance toward commercialization.

This review provides a structured and multi-dimensional framework that distinguishes it from previous summaries of self-healing strategies in perovskite solar cells. First, polymer-based self-healing approaches are categorized by substrate, especially whether it is a flexible or rigid PSCs. Then, it is further distinguished by the underlying bonding mechanism, separating the physical interactions from chemical dynamic bonds. Lastly, the perovskite composition is utilized to further categorize the review. By integrating these three dimensions, this review offers a systematic perspective on the usage of self-healing polymer for PSCs with emphasis on strengths, limitations, and future directions of regenerative polymer additives for integration to scalable PSCs and other perovskite optoelectronics.

## 2. Flexible PSCs

Flexible perovskite solar cells (FPSCs) recently emerged as a viable type of photovoltaic technology, providing unique benefits over rigid PSCs. Their lightweight and bendable characteristics have potential applications in aerospace, portable power sources, and building-integrated photovoltaics. Additionally, FPSCs have demonstrated comparable photovoltaic performances as their rigid counterpart, with the certified world record PCE of 24.90% as of this writing [12]. Unfortunately, although FPSCs have favorable features, they are not yet commercialized due to their limitations in mechanical durability, long-term operational stability, and environmental resistance. Under mechanical stress, hairline fractures form in the perovskite material that accumulate over time, compromising durability and performance in the long term [13]. As with all perovskites in both flexible and rigid PSCs, water molecules from humid environments also cause reactions with the perovskite, disassembling them into a degraded lattice structure and components [14]. As a result, many researchers focus on reducing these limitations through the addition of self-healing polymer to unlock the commercialization potential of FPSCs [15].

### 2.1. Physical Bonds

As mentioned previously, flexible perovskite solar cells suffer from mechanical stress due to their bending fatigue. To mitigate the limitation of FPSCs, many researchers incorporate polymer additives capable of forming physical bonds, particularly hydrogen bonding [16], to improve the mechanical stability of the device from stress-induced damage. Hydrogen bonds occur between an electronegative acceptor and a hydrogen donor. These bonds are typically introduced through functionalized perovskite precursors [17], interfacial layers [18], and polymer additives [19]. This interaction acts as a reversible passivation crosslink [20], passivates undercoordinated Pb^2+^ ions [21], and mitigates water infiltration due to its hydrophobic nature [22]. Compared to weaker intermolecular forces such as van der Waals interactions or general dipole–dipole attractions, hydrogen bonds offer a balance of sufficient bonding strength and reversibility, enabling more effective and reversible self-healing without compromising the structural integrity of the perovskite crystals. Their directionality also helps maintain structure during damage and repair cycles, making them particularly well-suited for use in FPSCs. In other words, the introduction of hydrogen bonds to FPSCs can improve the mechanical stability, mobility of charged carriers, and moisture resistance. This allows for the spontaneous rearrangement of polymer chains, incorporating self-healing properties to treated FPSCs. By integrating these physical bonding mechanisms into PSCs, researchers can develop more durable, efficient, and stable flexible solar cells capable of withstanding mechanical stress and environmental degradation while maintaining high photovoltaic performance.

Hydrogen bonding is essential to the self-healing mechanism of polymer additives for PSCs. These physical bond interactions enable the perovskite film to heal dynamically, reversing mechanical damage in films and elongating the lifespan of the resulting devices. Self-healing from hydrogen bonds utilizes the dynamic and reversible interactions between electronegative acceptors and hydrogen donors. These bonds are weaker than covalent bonds yet offer enough strength, around 10 to 40 kJ/mol, to break and re-form under ambient conditions [23]. When mechanical damage occurs, the hydrogen bonds at the fracture disperse without breaking the backbone of the polymer. Due to the intrinsic mobility of polymer chains, the functional groups participating in hydrogen bonding can dissociate, redistribute, and reform hydrogen bonding across the damaged region of fracture. This dynamic healing mechanism is energetically favorable, allowing the polymer to self-restore intermolecular cohesion [24].

Certain functional groups, such as thiourea, carboxyl, etc., are incorporated into the polymer chains, causing a favorable environment for strong hydrogen bonds to form. This results in the polymer cross-link network being able to dynamically reverse into its original state after mechanical stress [25]. As a result, the perovskite layer is capable of healing from mechanical cracks and the migration of ions. Additionally, hydrogen bonds also improve the adhesion of the polymer to the perovskite film and passivate surface defects on the film and grain boundaries, which results in improved photovoltaic performance of polymer-treated devices [26,27]. Due to these beneficial properties, implementing polymers that utilize hydrogen bonds into FPSCs has become an attractive area of research.

#### 2.1.1. Single-Cation Perovskites

To take advantage of the beneficial effects of polymer additives with hydrogen bonding, Finkenauer et al. tested a variety of mass concentrations of thiourea-triethylene glycol polymer (TUEG3) in MAPbI_3_ perovskite [28]. Mixed solutions of TUEG3 in perovskite (0.0%, 0.2%, 2.1%, 6.0%, and 16.6% concentration) were spin-coated to create perovskite thin films to test the advantages and consequences of different mass concentrations. The resulting device with a rigid structure of glass/ITO/PTAA/TUEG3-MAPbI_3_/PC_61_BM/BCP/Ag had a champion PCE of 17.4% at 0.2% TUEG3, and a flexible structure of PET/ITO/PTAA/TUEG3-MAPbI_3_/PC_61_BM/BCP/Ag had a champion PCE of 13.69% at 0.0% TUEG3. Although the addition of TUEG3 resulted in the successful integration of self-healing properties to the PSCs, it consequently caused a relatively lower PCE compared to the control devices with no addition of TUEG3.

Notably, TUEG3 contains thiourea groups (-NH-C(S)-NH_2_) with strong hydrogen bond donors and acceptors, and ether groups (-O-) can also engage in hydrogen bonding interactions, although weaker than thiourea groups. This causes TUEG3 to be able to reversibly break and reform to heal mechanical damages such as microcracks or fractures on the film. After 3000 cycles of bending, the device treated with TUEG3 preserved 94% of PCE and recovered 80% of lost PCE. In addition to self-healing abilities, TUEG3 passivates surface defects and those at grain boundaries, at the consequence of lower open circuit voltage and PCE at higher concentrations (6.0% and 16.6%). There is potential for improvement in both polymer engineering and the optimization of FPSCs to improve both the mechanical stability of the device and its efficiency.

Zhang et al. doped the FAPbI_3_ perovskite film with polysiloxane (PAT), which has thiourea hydrogen bonds, and the possibility of chelation with the perovskite enhanced the integrity of the perovskite lattice structure [29]. Additionally, the chelated lead ions and degradation products stabilize the perovskite structure and inhibit further breakdown of the perovskite film. The pyridine units in PAT passivated the grain boundary, increasing the mobility of charged carriers and reducing non-radiative recombination. Not only did the pyridine units act as surface passivants, but they also acted as Lewis bases to form chelation adducts with PbI_2_. This resulted in an enhanced grain size of the perovskite structure. The resulting device, which was fabricated at 40% RH with a p-i-n structure of PDMS/hc-PEDOT:PSS/PEDOT:PSS/PAT-FAPbI_3_/PC_61_BM/PEDOT:PSS/hc-PEDOT:PSS/PEI/Cu/PDMS encapsulation, had a PCE of 19.58% with self-healing ability and enhanced moisture stability. For instance, the doped FPSCs retained 85% of their original PCE after 800 bending cycles and displayed evidence of self-healing when stretched for 200 bending cycles at 20% strain. Additionally, improved moisture stability was seen when the doped PSCs preserved 83% of their initial PCE when exposed to 80% RH for 2000 h due to the hydrophobicity of the siloxane group in PAT.

One of the most significant advantages of PSCs is the capability to be cost-effectively manufactured using a roll-to-roll printing mechanism. As a result, Kang et al. synthesized an ionogel (IG) to optimize FPSCs for roll-to-roll printing [30]. Hydrophobic fluorinated acrylic acid with acrylamide was copolymerized to create a polymer network with dynamic hydrogen interactions to add self-healing properties. The addition of ionogel to the perovskite film enhanced the resistance of the device to bending. The IG-treated and control FPSCs were bent with a 5 mm radius for 25,000 cycles. After the bending, the IG-treated device retained more than 90% of the initial PCE compared to the control device, which lost the majority of its PCE. The device morphology is significantly better for the IG-treated device than the control after bending fatigues due to the room temperature self-healing property of IG.

The moisture stability is enhanced with the addition of IG due to the hydrophobic trifluoromethyl groups in the polymer network. The perovskite film of the unencapsulated control device almost completely degraded after being exposed to water droplets for 2 s, while the film of the IG-added PSC only started decomposing after 60 s. This is significant because it allows FPSCs to roll-to-roll print without humidity control. The resulting device with the structure of flexible substrate/indium tin oxide (ITO)/poly[bis(4-phenyl)(2,4,6-trimethylphenyl)amine](PTAA)/IG-MAPbI_3_/choline chloride/C_60_/bathocuproine (BCP)/Cu had a PCE of 21.76%.

#### 2.1.2. Double-Cation Perovskites

Many groups synthesize their polymer additive to have strong interactions with the perovskite crystals. For example, Zhang et al. synthesized polysiloxane (SHP) and used it to dope FAMAPbI_3_ perovskite in order to attain self-healing properties [31]. This specific polymer was synthesized for its dynamic 2,6-pyridinedicarboxamide (PDCA) coordination units and hydrogen bonding capabilities. The self-healing property of SHP due to abundant dynamic hydrogen bonds was seen when SHP-treated FPSCs recovered 80% of their initial PCE after being strained 1–30% for 150 cycles. The coordination units interact with lead and iodine ions on the perovskite film, reducing trap densities and improving the charged carrier mobility and grain sizes. Not only did SHP passivate the perovskite film and improve the PCE of the treated PSCs, but the hydrophobic property of SHP from siloxane also improved the moisture stability with 82% normalized PCE when exposed to 20% RH for 3000 h.

**Figure 1:**
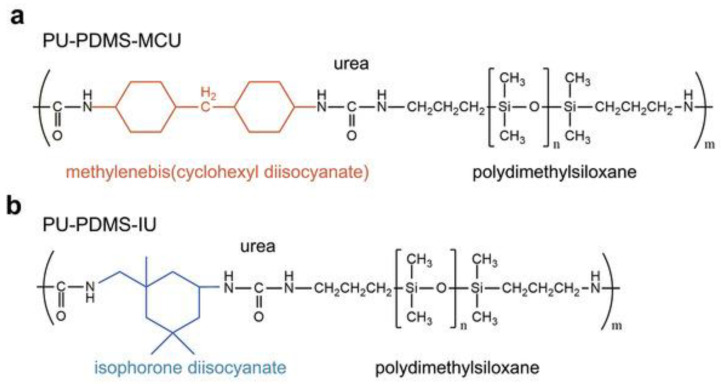
(**a**) The figure displays the molecular structure of PU-PDMS-MCU. This polymer has methylenebis (cyclohexyl diisocyanate), urea, and polydimethylsiloxane groups. (**b**) The figure illustrates the molecular structure of PU-PDMS-IU. This polymer has isophorone diisocyanate, urea, and polydimethylsiloxane groups [32].

Additionally, Han et al. synthesized two types of polyurea (PU) with polydimethylsiloxane (PDMS) blocks, which are associated with two different diisocyanate units: 4,4′-methylenebis(cyclohexyl diisocyanate) unit (MCU) and isophorone diisocyanate unit (IU), as can be seen in Figure 1 [32]. The resulting polymers, PU-PDMS-MCU and PU-PDMS-IU, are chosen for their specific chemical properties: self-healing abilities of the elastomer chains, hydrophobicity of PDMS, rigidity and abundance of hydrogen bonding capabilities of the cyclic diisocyanate units, thermal stability of the overall polymer, and surface passivation abilities through Lewis acid-base interaction with the perovskite.

**Figure 2:**
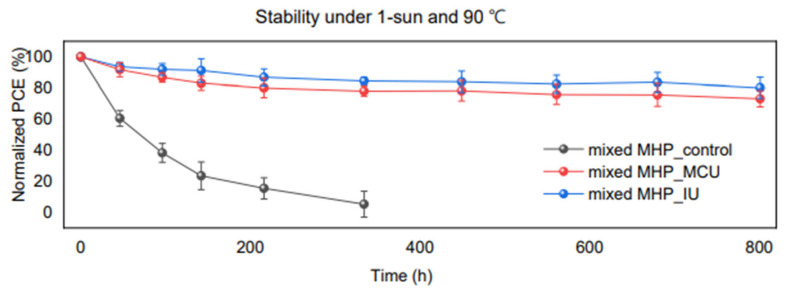
The graph displays the results from thermal and operational stability tests of the control device, PU-PDMS-MCU-treated device, and PU-PDMS-IU-treated device for 800 h under 1 sun at 90 °C. The control device lost most of its initial PCE before 400 h, while the polymer-treated devices retained more than 80% of their initial PCE for 800 h [32].

The resulting device with the structure of PEN/PEDOT:PSS/PTAA/MA_0.25_FA_0.75_Pb(Cl_0.6_I_2.4_)/PU-PDMS-IU/PC_60_BM/BCP/Au had improved self-healing abilities, operational stability, and thermal stability compared to the control. The addition of PU-PDMS-IU to FPSCs introduced a self-healing ability, which is shown in the flexibility testing. The polymer-treated device and control were bent at a 2 mm radius, or 1.29% mechanical strain, and the control retained only 15% of its original PCE after 1000 bending cycles. In contrast, the PU-PDMS-IU-treated device maintained roughly 90% of its initial PCE after 2000 bending cycles. Additionally, as can be seen in Figure 2, the thermal and operational stability of PU-PDMS-IU-treated devices was greatly improved. This is due to the polymer’s strong interaction with the perovskite crystals, which suppressed defect migration and passivated surface defects.

Not all polymer additives have their self-healing mechanism triggered at room temperature. For example, Ge et al. utilized a supramolecular adhesive (AD-23) to improve the mechanical stability of FPSCs [33]. AD-23 was synthesized through a random copolymer synthesis involving acrylamide (AM) and n-butyl acrylate (BA). This material is capable of thermal self-healing due to its amphiphilic properties and the abundance of hydrogen-bond donors and acceptors. Heating is required to induce movement of molecular chains that causes uniform re-distribution of hydrogen bonds, which results in self-healing.

**Figure 3:**
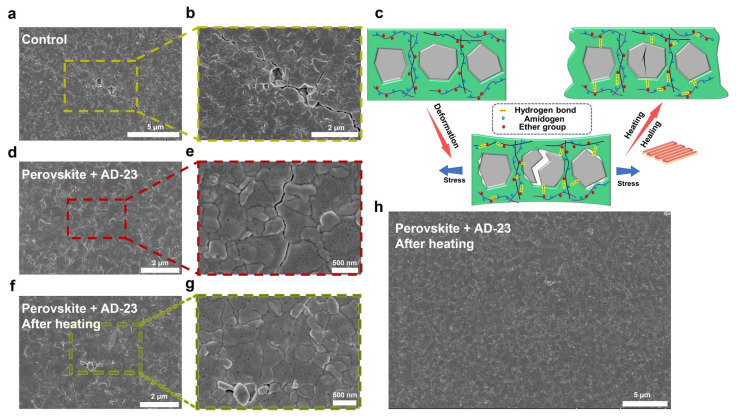
Images (**a**,**b**) are from a scanning electron microscope of control FPSCs after the bending test. Illustration (**c**) demonstrates the self-healing mechanism of the polymer additive through hydrogen bonds. SEM images (**d**,**e**) are AD-23-treated FPSCs after the bending test. SEM images (**f**–**h**) are AD-23-treated FPSCs after the bending test and thermal healing at 70 °C for 5 min [33].

AD-23-treated and control FPSCs were bent with a 1.5 mm radius for 2000 cycles, which induce cracks along the grain boundary. The AD-23-treated FPSCs are heated to a relatively low temperature of 70 °C for 5 min to trigger the thermal self-healing. Scanning electron microscope (SEM) images were taken, and images of the AD-23-treated devices displayed that the film was evenly repaired, as shown in Figure 3.

**Figure 4:**
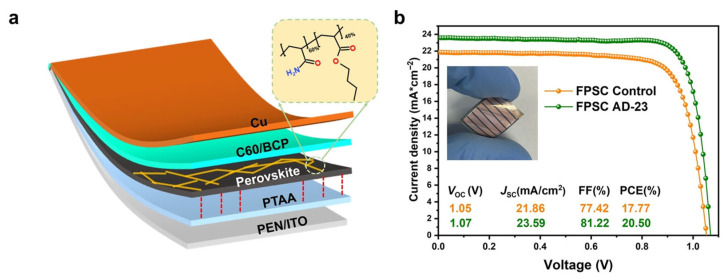
(**a**) The illustration displays the device structure of FPSC with the following layers: PEN/ITO/PTAA/FAMAPbI_2_ + AD-23/C_60_/BCP/Cu. (**b**) The graph shows the J-V (current density-voltage) test of FPSC: the control device is shown in orange, and the device with AD-23 is shown in green. The open-circuit voltage (V_OC_), short-circuit current density (J_SC_), fill factor (FF), and power conversion efficiency (PCE) are all higher for the flexible device with AD-23 compared to the control [33].

The addition of AD-23 also greatly enhanced the photovoltaic performance of FPSCs, as AD-23 modules passivate surface defects and lower trap state density, which improved the mobility of charged carriers. As shown in Figure 4, the resulting device with the structure of PEN/ITO/PTAA/FAMA PbI_3_ + AD-23/C_60_/BCP/Cu had a PCE of 20.50% with higher stability and efficiency than the control when aged at 85 °C and 85 % to 96% relative humidity (RH) for 260 h.

#### 2.1.3. Triple-Cation Perovskites

To address both the lifespan and efficiency of FPSCs, Yang et al. utilized two polymers, glycidyl methacrylate-bonded β-cyclodextrin (GMA-CD) and N-adamantylacrylamide (N-AA), and a surface passivant, methacryl guanidine hydrochloride (MAGH), to improve the photovoltaic performance of FPSCs [34]. The surface passivant, MAGH, formed a 2D/3D heterostructured perovskite film to reduce trap densities on the surface of perovskite and improve the stability of the resulting device. The resulting PSCs with the structure of PEN/FTO/SnO_2_/Cs_0.05_(MA_0.11_FA_0.89_)_0.95_Pb(I_0.97_Br_0.03_)_3_ with MAGH, GMA-CD, and N-AA/Spiro-OMeTAD/Ag demonstrated a decent efficiency over 23% on glass substrate and a PCE of 20.46% for FPSCs.

The dynamic hydrogen bonding interaction between GMA-CD and N-AA, which further interacts with the perovskite, improved the mechanical stability of FPSCs due to the introduction of self-healing. When bent at a radius of 6.25 mm, the control device retained 60% of its original PCE after 2000 bending cycles, while the treated device maintained more than 85% of its initial PCE after 4000 cycles. Not only did the polymer additives result in the perovskite film being able to self-heal mechanical damage, but the 2D/3D perovskite structure from the addition of MAGH resulted in enhanced moisture stability. Interestingly, when exposed to 60–70% RH for 4 h after enduring 4000 bending cycles, the treated FPSCs recovered over 90% of their initial efficiency.

In short, the mechanical stability of FPSCs is enhanced with the usage of self-healing polymer additives with hydrogen bonds. The dynamic and reversible hydrogen bonding repairs microcracks and defects formed during the operation of FPSCs, resulting in enhanced long-term operational durability of FPSCs. Many researchers utilize various hydrogen-bond-based polymers, such as TUEG3, AD-23, IG, GMA-CD, N-AA, SHP, PU, PU-PDMS-MCU, and PU-PDMS-IU, to leverage hydrogen bonding interactions to facilitate self-healing of fractures of perovskite film. By integrating these polymers with hydrogen bonding and interactions with perovskite to FPSCs, researchers have significantly enhanced both the mechanical and operational stability of devices.

### 2.2. Chemical Bonds

Although physical bonds typically interact quickly and reversibly to trigger healing at ambient conditions, they often do not contribute to long-term stability and mechanical strength. Since physical bonds mostly consist of forces and interactions that require minimal energy input, the energy required to separate them is equally low. In contrast, a self-healing mechanism using chemical bonds, precisely, dynamic covalent bonds, enhances durability and introduces a stimuli-induced repairing mechanism. Although covalent bonds require greater energy input than physical bonds, the requirements for these bonds to form are produced easily enough to remain a viable mechanism for polymer-based self-healing in FPSCs. For instance, a dynamic disulfide bond is an emerging type of covalent interaction that self-heals under a mild stimulus, such as UV irradiation [35] or thermal treatment with heat [36]. Many researchers use heat treatment as the stimulus to induce this self-healing mechanism, which is favorable in applications to FPSCs because often the temperatures required for self-healing are within operational conditions of solar cells. As a result, the use of polymer additives with disulfide bonding has become a significant area of study for researchers, with a large focus being placed on turning unfavorable environmental conditions into regenerative conditions for the FPSCs.

Disulfide-based self-healing polymer additives are utilized by many researchers due to their dynamic covalent bonds. The reversible sulfur–sulfur (-S-S-) bonds allow the polymer to autonomously repair itself after environmental or mechanical degradation. These bonds are often formed through oxidative coupling with thiol groups (-SH). Due to their moderate bond dissociation energy of around 250 kJ/mol, these bonds are capable of breaking and reforming under mild conditions, such as thermal, photochemical, or redox stimuli [37]. Compared to hydrogen bonds, disulfide-based covalent bonds are stronger and more effective for self-healing, which usually requires external activations, such as thermal or light energy. Under thermal conditions, which is the most common way researchers initiate disulfide-based self-healing, disulfide bonds undergo homolytic cleavage to form reactive thiyl radicals, which reassemble across damaged areas to reconstruct the polymer network. Similarly, light exposure, such as UV rays, induces similar radical-involved exchange, while redox stimuli cause a nucleophilic substitution through thiol-disulfide exchange reactions [38]. In the context of self-healing, the mobility of the polymer chain is essential as it allows fragments to disperse, rearrange, and reform disulfide crosslinks at fracture interfaces.

Offering greater strength at the cost of required energy input, disulfide bonds demonstrate notably larger contributions towards the self-healing properties of perovskite [39]. This disulfide covalent bond-based strategy can result in the mitigation of long-term PCE degradation from mechanical stress and unideal environmental conditions.

#### 2.2.1. Single-Cation Perovskites

Not only is polyurethane (PU) a popular choice for hydrogen-bonding-based self-healing polymer additives for FPSCs, but PU with disulfide bonds is also used by many researchers to improve the mechanical stability and lifetime of FPSCs. For example, Lan et al. utilized polyurethane elastomers with disulfide bonds (PUDS) as a self-healing polymer for FPSCs [40]. Disulfide bonds break at temperatures beyond 60 °C, forming free radicals and reforming disulfide bonds when temperatures are cooled below 60 °C. Additionally, the carbonyl group in PUDS interacts with the Pb^2+^ of the perovskite film, increasing the grain size and crystallinity of the perovskite film. The resulting FPSCs with the structure of PEN/ITO/NiOx/MAPbI_3_ + PUDS/PC_61_BM/PEI/Ag had a PCE of 17.19%. Additionally, PUDS-treated FPSC retained 95% of its initial PCE when stored in the N_2_ glovebox for 3000 h.

Most importantly, the dynamic disulfide bonds of PUDS resulted in the improved mechanical stability of FPSCs. After 4000 bending cycles at 3 mm radius, PUDS-treated FPSC retained 84% of the initial PCE. Furthermore, to test the self-healing ability of PUDS, polymer-treated FPSC was cracked at a bending radius of 2 mm to have visible mechanical damage. This device was exposed to continuous heat treatment at 80 °C for 10 min, and after the heat treatment, the PCE recovered from 12% to 88% of the initial PCE.

#### 2.2.2. Triple-Cation Perovskites

**Figure 5:**
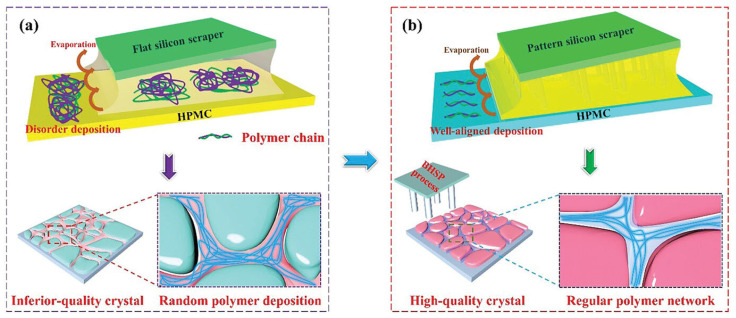
Deposition behavior of polymer chains during the meniscus coating process. (**a**) Random polymer deposition typically observed in conventional printing methods. (**b**) Oriented polymer growth along the transparent electrode and perovskite grain boundaries was achieved through the bionic high-speed patterned (BHSP) meniscus coating technique [41].

Similarly, Gong et al. coordinated PU with disulfide bonds specifically on the perovskite active layer to ensure that it is oriented to take advantage of the benefits of the self-healing polymer additives [41]. As seen in Figure 5, this group used patterned-meniscus coating technology to add PU in the perovskite film, as the uncoordinated deposition of polymer chains on the perovskite film can cause an interference with the movement of charged carriers. PU was specifically chosen to improve the bending resistance of FPSC, as PU has a disulfide bond. The crystallinity of the perovskite film was improved as PU interacted with the Pb^2+^ ions of perovskite.

The resulting device with the structure of HPMC/hc-PEDOT:PSS/SnO_2_/CsFAMA/PU/Spiro-OMeTAD/Ag had a PCE of 20.04%. It also had improved moisture stability as it maintained 91% of its original efficiency after being exposed to air for 3000 h and mechanical stability as it retained 86% of its original PCE after 1000 bending cycles at a stretch ratio of 30%. Additionally, polymer-treated FPSC was bent at a 3 mm radius and treated with heat to demonstrate the self-healing property of the polymer additive. There were visible mechanical cracks along the perovskite film from the bending. After being exposed to continuous heat treatment at 60 °C for 1 h, the PU repaired the fractured grain boundaries of the perovskite film.

To summarize, the incorporation of polymers that utilize disulfide bonds can result in the performance restoration and significant reduction in defects and damage caused by mechanical stress and environmental factors. With heat treatment, disulfide polymers can break down and distribute into damaged regions before reforming to greatly reduce long-term damage and photovoltaic performance loss. As a result of its ease of customization for specific purposes and its ideal properties for use in perovskites, the polyurethane family of materials is the preferred choice of many researchers. By incorporating it into PSC films, long-term efficiency and durability can be maintained.

### 2.3. Both Physical and Chemical Bonds

While both hydrogen and covalent disulfide bond-based polymers individually serve as efficient methods for performance recovery in PSCs, polymers that take advantage of both physical hydrogen bonds and chemical bonds are shown to have an even greater effect. Requiring less initial energy and maintaining a higher percentage of PCE over long durations of time, these polymers provide greater results in device performance and self-healing in comparison to their single-bond type counterparts. By reforming strong covalent bonds under high temperatures and passive hydrogen bonds over time, these treated PSCs are capable of both rapid short-term performance recovery and long-term passive restoration of cracks. As a result, the PSCs are able to be healed more efficiently under a wider range of environmental conditions. Table 1 summarizes and compares the characteristic timescales and energy requirements, highlighting how low-energy hydrogen bonding contributes to fast initial recovery while disulfide exchange enables slower, long-term network repair. Hydrogen bonds reform rapidly with low energetic barriers, enabling fast initial crack closure, whereas disulfide exchange proceeds more slowly and requires higher activation energy, supporting long-term network rearrangement and permanent mechanical recovery.

#### 2.3.1. Single-Cation Perovskites

**Figure 6:**
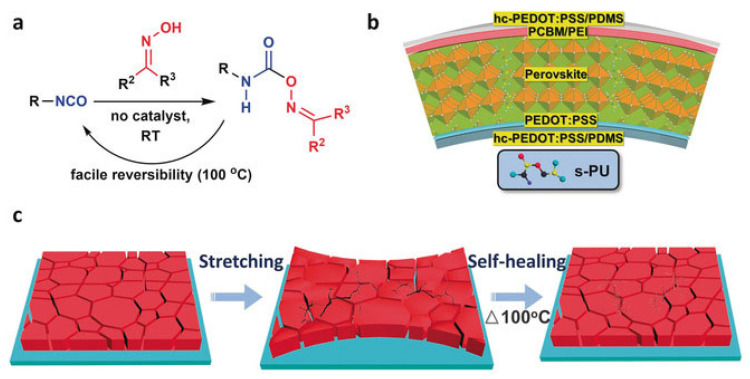
(**a**) The synthesis of PU. (**b**) The device structure treated with s-PU. (**c**) The illustration displays the process of s-PU-treated perovskite film being stretched, resulting in fractured film. Then, the film is thermally annealed at 100 °C to self-heal the mechanical damage [42].

Some researchers use other types of dynamic covalent bonds rather than disulfide bonds with non-covalent hydrogen bonds to promote self-healing in FPSCs. For instance, Meng et al. synthesized self-healing polyurethane (s-PU) with hexamethylene diisocyanate and multifunctional oximes [42]. s-PU contains dynamic covalent oxime-carbamate bonds, which act as scaffolds in the perovskite film, not only serving as self-healing agent, but also improving grain size while passivating both surface and boundary defects. When s-PU-treated FPSCs are under mechanical stress, the s-PU with flexibility absorbs some of the stress until the perovskite grain boundaries are broken to further release the mechanical force. The cracked-s-PU is easily self-healed when thermally annealed at 100 °C, which is shown in Figure 6.

**Figure 7:**
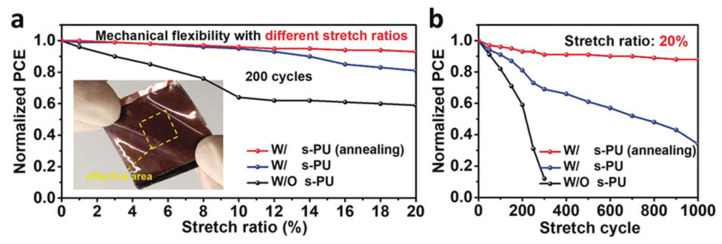
(**a**) A graph from the mechanical flexibility test where devices, including control device, with s-PU, with s-PU and annealed to initiate thermal self-healing, were stretched to different stretch ratios from 0 to 20%. The results show that the normalized PCE is significantly better for devices with s-PU and even better for the device that was thermally annealed with s-PU. (**b**) A graph from the mechanical stability test where control, s-PU-treated, and thermally annealed s-PU-treated devices were stretched for 1000 bending cycles. The normalized PCE of the thermally annealed s-PU-treated device was noticeably higher than the control and non-thermally annealed s-PU devices [42].

As a result, the mechanical stability of s-PU-treated FPSCs is greatly enhanced: polymer-treated FPSCs retained 88% of their initial PCE after 1000 cycles of bending at 20% stretch, as seen in Figure 7. This increased stability against mechanical stress can be attributed to the covalent oxime-carbamate bonds and non-covalent hydrogen bonds that reform after fractures due to mechanical force.

Not only is the mechanical stability increased with the addition of s-PU to FPSCs, but humidity stability is increased as well. Due to the self-encapsulating structure of the upper PDMS layer, the moisture stability of the s-PU-treated device greatly improved. For example, the polymer-treated device retained 90% of its initial PCE when stored in atmospheric conditions at 40% RH. The resulting device with the structure of PDMS/hc-PEDOT:PSS/PEDOT:PSSAl4083/MAPbI_3_/PCBM/polyethyleneimine(PEI)/hc-PEDOT:PSS/PDMS had a PCE of 19.15%. Because reproducibility is crucial for practical application, the steady state efficiency and current density were measured over 600 h under 1 sun illumination; a continuous efficiency of 19.11% was observed.

#### 2.3.2. Double-Cation Perovskites

**Figure 8:**
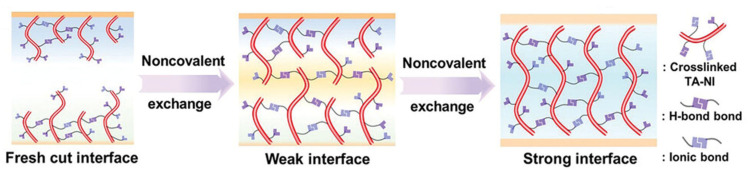
A schematic illustrating the self-healing mechanism of cross-linked TA-NI at room temperature [43].

Chen et al. synthesized cross-linkable monomer 5-(1,2-dithiolan-3-yl) pentanehydrazide hydroiodide (TA-NI) into a cross-linked polymer network [43]. The covalent disulfide bonds and non-covalent hydrogen bonds allow the polymer to have elastomeric properties, enabling self-healing of mechanical damage at room temperature, as seen in Figure 8. 

The cross-linking polymer acts as ligaments anchored to the grain boundaries of the perovskite film, which consists of 1D perovskite and elastomers. The ammonium reacts with the PbI_6_^4−^ in 3D FA_0.92_MA_0.08_PbI_3_ perovskite film and forms a 1D/3D heterostructured perovskite film. The 1D perovskite passivates the surface and grain boundary of the 3D counterpart while also acting as a protective layer against moisture-induced degradation. The resulting device with the structure of polyethylene terephthalate (PET)/ITO/SnO_2_/FA_0.92_MA_0.08_PbI_3_/Spiro-OMeTAD/Au had a PCE of 21.66% with enhanced mechanical, operational, and ambient stabilities. In three combined test conditions, the TA-NI-treated FPSCs retained 90% of their initial PCE after 20,000 bending cycles at 5 mm radius, exposure to 1 sun illumination at 44–55 °C for 1248 h, and storage at ambient atmosphere with an RH of 30% for 3000 h.

**Figure 9:**
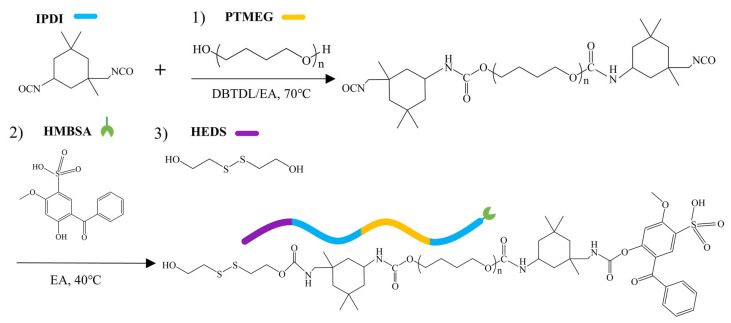
The stepwise synthesis of the modified polyurethane adhesive (PUA) is shown. IPDI reacts with PTMEG to form a urethane prepolymer, which is then extended with HMBSA, and finally functionalized with HEDS to introduce the dynamic sulfide bonds [44].

Based on similar FAMA perovskite (FA_0.8_Cs_0.2_PbI_3_), Zhu et al. synthesized a modified polyurethane adhesive (PUA) from the reaction of polyisocyanate and polyol with a catalyst [44]. The reversible covalent disulfide bonds and non-covalent hydrogen bonds are reformed at the fractured film, which facilitates the self-healing of the film. As shown in Figure 9, the asymmetric alicyclic structure of isophorone diisocyanate (IPDI), a reactant in the synthesis of PUA, greatly benefits the self-healing process, as it enhances the polymer chain mobility. Due to the presence of covalent and non-covalent bonds mentioned above, PUA-treated FPSCs can self-heal at room temperature within 45 min. These devices can be heated to temperatures ranging from 25 °C to 85 °C to expedite the process: the higher the temperature, the quicker the self-healing is.

Not only is PUA used as a self-healing polymer additive in perovskite, but PUA film is also coated on the bottom metal electrsode to encapsulate the device via direct conglutination of the substrate. This results in an enhanced lifetime of the resulting FPSCs: PUA-encapsulated devices retain 92.6% of their original PCE for 1825 h when stored at 20% RH and room temperature of 25 °C. The resulting flexible modulus (6.5 cm × 6.5 cm) on polyethylene terephthalate (PET) substrate and with FA_0.8_Cs_0.2_PbI_3_ perovskite had a PCE of 17.2%.

Another dynamic covalent bond utilized by researchers is the acrylhydrazone bond. For example, Xu et al. synthesized acylhydrazone-bonded waterborne polyurethane (Ab-WPU), which can self-heal mechanical damages of FPSCs at operational conditions [45]. The acrylhydrazone bond in Ab-WPU is a type of dynamic covalent bond and can reverse mechanical damage on perovskite film at 60 °C. The mild temperature of 60 °C is in the typical temperature range solar cells experience under continuous sun illumination, which enables Ab-WPU-treated FPSCs to self-heal at operating conditions.

In addition to a self-healing property for FPSCs, the Ab-WPU additive regulates the crystallization of the perovskite film when Ab-WPU is added to the perovskite precursor solution. As a result, Ab-WPU can be utilized as a Pb^2+^ ion chelating agent to improve the crystallinity of the perovskite film. In addition to the improved crystallinity of the perovskite film, Ab-WPU also passivates surface defects on the perovskite film. The polymer consists of carbonyl functional groups, which can interact with cationic undercoordinated ions, such as FA^+^ and Pb^2+^. This surface defect passivation reduces trap densities and increases the mobility of charged carriers. The resulting device with the structure of PEN/ITO/MeO-2PACz/FA_0.87_Cs_0.13_PbI_2.7_Br_0.3_/PC_61_BM/BCP/Ag had a PCE of 21.27% with enhanced moisture, thermal, and mechanical stabilities. The polymer-treated FPSC was bent for 1000 cycles at a 6 mm radius and retained 95% of its initial PCE after a 30-min thermal treatment on a hotplate at 60 °C.

#### 2.3.3. Triple-Cation Perovskites

**Figure 10:**
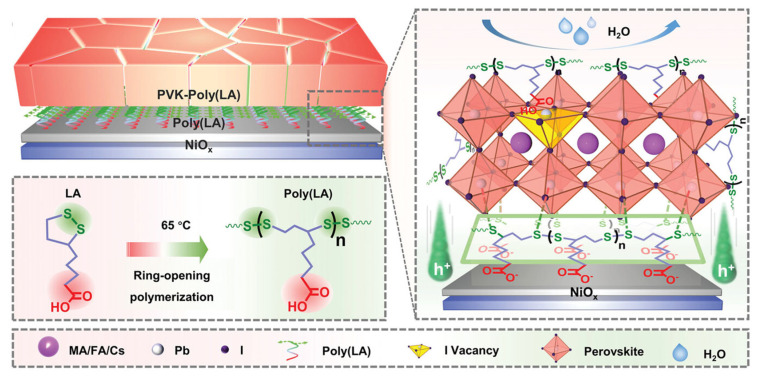
The illustration demonstrates the thermal-initiated ring-opening polymerization of LA into Poly(LA) at 65°C and the interface optimization of NiOx and perovskite by the addition of Poly(LA) [46].

Many researchers utilize a polymer additive with both hydrogen bonds and dynamic covalent disulfide bonds to realize self-healing capability. For example, Yang et al. cross-linked α-lipoic acid (LA) into polymerized LA, or Poly(LA), which has non-covalent hydrogen bonds of carboxyl groups and dynamic covalent disulfide bonds [46]. Poly(LA) can self-heal after a mild heat treatment of 65 °C. When the temperature exceeds its melting point of 65 °C, a thermally catalyzed ring-opening polymerization of the disulfide bond happens in Poly(LA), as seen in Figure 10. Under mechanical stress, the disulfide bond, intermolecular hydrogen bonds, and other interactions are disconnected, resulting in fractures of the perovskite film. Then, the hydrogen bonds reconstruct, and the disulfide bond rearranges when exposed to 65 °C heat treatment.

**Figure 11:**
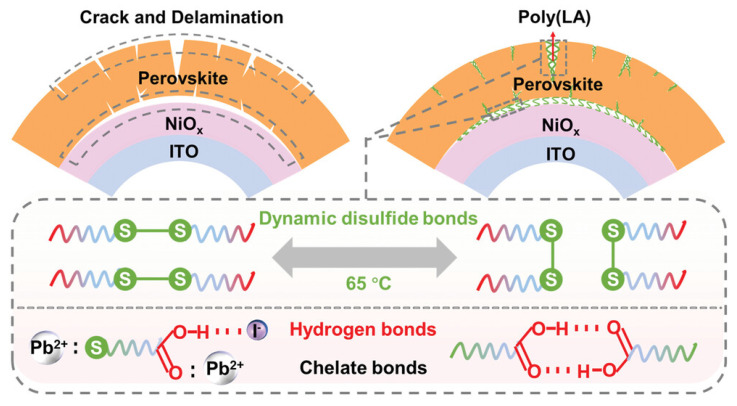
Schematic illustration showing the formation of cracks and delamination due to mechanical bending, along with the self-healing mechanism of the optimized device enabled by dynamically cross-linked Poly(LA) networks incorporating disulfide bonds, hydrogen bonds, and chelate interactions [46].

In addition to the self-healing properties, the hydrogen bonds of Poly(LA) interact with undercoordinated Pb^2+^ ions of perovskite film to passivate surface defects and improve the crystallinity of the resulting film. In consequence, the resulting device with the structure of PET/ITO/NiOx/Poly(LA)/CsFAMA-Poly(LA)/PC_61_BM/BCP/Ag had a PCE of 19.03% with improved mechanical and thermal stability. For instance, after being exposed to continuous bending cycles, control and Poly(LA)-treated FPSCs were annealed at 65 °C for 6 h. The SEM results from this bending test, shown in Figure 11, display that cracks on the perovskite film are healed, where it is almost visually identical to the original film prepared without mechanical force applied. Additionally, the polymer-treated device retained 95% of its initial PCE after the heating treatment.

Not only is the self-healing of Poly(LA) efficient at a mild temperature of 65 °C, but it is also able to self-heal at room temperature over a long period of time. After the same previously mentioned bending test, the polymer-treated device stored at room temperature recovered device performance, which indicates that the non-covalent hydrogen bond enables the Poly(LA)-treated device to self-heal at lower temperatures than the mild temperature point of 65 °C. After 3000 bending cycles at 5 mm radius, the control device had an 80% decrease in PCE from its initial value; meanwhile, the Poly(LA)-treated device maintained more than 80% of its original PCE.

Similarly, Yang et al. synthesized poly(dimethylsiloxane) polyurethane (DSSP-PPU) with disulfide bonds and multiple hydrogen bonds: urea-ethyl carbamate, urea-urea, urea-ethyl carbamate, and N^2^,N^6^-bis(2-hydroxyethyl)-2,6-pyridinedicarboxamide (BHP)-BHP [47]. The presence of dynamic covalent disulfide bond exchange and multiple hydrogen bonds in the polymer enables room-temperature reformation of non-covalent and covalent bonds at the cracked interface. For example, scratches on DSSP-PPU film are healed in 30 min at room temperature, and an entirely fractured film is self-healed within an hour at room temperature if the two films are in contact with each other. Additionally, hydrogen bonds can break to release the stress from mechanical force, enhancing the flexibility of the resulting FPSCs. For instance, when bent at a 2 mm radius for 8000 bending cycles, the DSSP-PPU-treated FPSC with the structure of PET/PEDOT:PSS PH1000/poly(triaryl amine)/Al_2_O_3_/Cs_0.05_MA_0.10_FA_0.85_Pb(I_0.97_Br_0.03_)_3_/PCBM/BCP/Ag retained 80% of its original PCE. After the self-healing process, the PCE was further recovered to nearly 90% of its initial efficiency. For bending testing, PET/PEDOT:PSS PH1000 rather than ITO was used due to the fragile nature of ITO. In efficiency testing ITO was used. The resulting device with the structure of polyethylene naphthalate (PEN)/ITO/SnO2/Cs_0.05_MA_0.10_FA_0.85_Pb(I_0.97_Br_0.03_)_3_/Spiro-OMeTAD/Ag had a PCE of 22.24%.

In a similar fashion, Chen et al. synthesized PAB, a self-healing polysiloxane elastomer, to enhance the ambient stability of FPSCS [48]. PAB was created through a thermally initiated stepwise polymerization of bis(3-aminopropyl)-terminated poly(dimethylsiloxane) (PDMS), isophorone diisocyanate (IPDI), bis(4-aminophenyl) disulfide (AFD), and 4,4′-bis(hydroxymethyl)-2,2′-bipyridine (BHB). The PDMS is the flexible, hydrophobic backbone, IPDI provides urethane and amide units for hydrogen bonds, AFD introduces dynamic covalent disulfide bonds, and BHB incorporates bipyridine coordination sites for the perovskite to interact with and additional hydrogen bonds.

The perovskite precursor solution was doped with PAB, which resulted in the PAB localizing mainly at the grain boundary. This led to the self-healing at the grain boundary of perovskite film through breakage and recombination of hydrogen bonds and disulfide bonds, which also had a supplementary effect of surface defect passivation. The hydrophobic property of PAB increased the overall environmental stability of the perovskite film, which is shown in the high performance and stability of ambient-air fabricated films. The resulting device with the structure of PEN/FTO/SnO_2_/PAB-Cs_0.05_FA_0.85_MA_0.10_Pb(I_0.97_Br_0.03_)_3_/Spiro-OMeTAD/Au had a reduced Young’s modulus of 4.82 MPa, as compared to modulus of 7.77 MPa from the control film without PAB doping. Additionally, after 200 bending cycles at 20% strain, the SEM images of the PAB doped device showed no signs of interface delamination, while the control device without doping showed signs of interface dysconnectivity.

Particularly, suppression of defect and recombination was through the coordination of bipyridine in PAB and Pb^2+^ ion of the perovskite, which was confirmed by density functional theory (DFT) calculations. This coordination reduces the amount of uncoordinated lead ions, thereby decreasing trap density and improving charge transportation. The resulting rigid PSCs fabricated for measuring device efficiency, with a structure of glass/FTO/SnO_2_/PAB-Cs_0.05_FA_0.85_MA_0.10_Pb(I_0.97_Br_0.03_)_3_/Spiro-OMeTAD/Au, demonstrated PCE of 21.02% and fill factor of 81.18%.

In conclusion, FPSCs with polymers that utilize both physical and chemical bonds can produce results comparable to those with just chemical bonds, but with a fraction of the required energy due to hydrogen bonds. At room temperature, many of these polymers can still greatly improve the regenerative properties of perovskite. Additionally, these polymer-treated PSCs demonstrate greater stability in humid environments. However, as a consequence of having greater complexity, these polymers have to be curated for compatibility with other compounds used in PSC development. As a result, some PSCs developed using hydrogen and covalent disulfide bond-based polymers can demonstrate lower PCEs due to their focus on maintaining stability. Despite this compromise, these polymer-treated PSCs still boast a respectable PCE that far outpaces efficiency-focused devices in the long run. Further research in self-healing polymers with both physical and chemical bonds to improve the PCE while maintaining durability could prove beneficial for the future commercialization of FPSCs.

## 3. Rigid PSCs

In contrast to FPSCs, rigid perovskite solar cells offer no elasticity but superior long-term mechanical stability. Rigid PSCs are preferred in the traditional application of solar cells where the primary considerations are long-term operational stability and efficiency, rather than light weight and flexibility [49]. The current research in this area focuses on improving the photovoltaic performance of PSCs through surface passivation [50], encapsulation [51], modified interfacial layers [52], and the addition of self-healing polymers. Since rigid PSCs are typically not placed at the position under the same mechanical stress as FPSCs, the focus of implementing polymers has been on protecting against environmental conditions, such as high humidity, that reduce stability and efficiency over time. Some of this research has found self-healing polymers capable of taking advantage of typically unideal conditions and using the energy provided and processes involved in them to induce self-healing [53]. Additionally, the compatibility of polymers with other components of the PSC devices is greater than in FPSCs, which leads rigid PSCs to have a higher PCE ceiling than their flexible counterparts.

### 3.1. Physical Bonds

As mentioned previously, the primary concerns for rigid PSCs are their long-term operational stability and efficiency. To mitigate the limitation of PSC durability, many researchers incorporate polymer additives capable of forming physical bonds, which, for self-healing polymers as discussed earlier, is generally limited to hydrogen bonds due to their relative high strength when compared to dipole–dipole or van der Waals forces [54]. Oftentimes, hydrogen bonding interactions enhance the stability of the device, especially moisture stability, through their ability to reverse moisture-induced degradation. The polymer additives are typically incorporated into the device through doping of the perovskite layer by adding monomers to the perovskite precursor solution. The interaction between the polymer added and the perovskite structure anchors ions of perovskite when degraded from environmental conditions, such as moisture, and later reverses to the original state due to the dynamic nature of hydrogen bonds. Additionally, the introduction of hydrogen bonds can passivate surface defects to further improve the long-term stability, mobility of charged carriers, and efficiency of the resulting device [55,56,57,58,59,60]. By integrating physical bonding mechanisms into PSCs, researchers can develop more durable, efficient, and stable rigid PSCs capable of withstanding environmental degradation while retaining high photovoltaic performance.

Similar to FPSCs, hydrogen bonding serves a critical role in the self-restorative properties of rigid PSCs. Unlike in FPSCs, where the focus of the self-healing mechanism was restoring mechanical cracks, hydrogen bonding interactions in rigid PSCs focus on dynamically stabilizing degradation intermediates and allowing them to reintegrate into the perovskite lattice to repair from moisture-induced degradation [61]. Under humid conditions, the perovskite structure partially decomposes. However, if the polymer additive contains hydrogen bond acceptors or donors, it can form reversible, dynamic hydrogen bonds with the cations of degradation intermediates, such as MA^+^ or FA^+^. This anchors the cations to the grain surface and prevents the loss of these intermediates. Once the moisture is removed, the localized cations reintegrate into the perovskite structure, as they are within proximity to react within the perovskite structure to spontaneously recombine and regenerate the lattice structure. As a result, the perovskite layer is capable of healing from high humidity environment. Additionally, hydrogen bonds also improve the adhesion of polymer to the perovskite film and passivate surface defects on the film and grain boundaries, as well as defects on the interface between perovskite and hole transport layer (HTL), which results in improved photovoltaic performance of polymer-treated devices [62,63,64].

#### 3.1.1. Single-Cation Perovskites

Wang et al. doped FAPbI_3_ perovskite film with a multifunctional starch-I_2_ complex to increase the quality and stability of the film [65]. The complex releases I_2_ to oxidize the Spiro-OMeTAD HTL, which improves the quality of the resulting perovskite film and PSC fill factor. For instance, the device with starch-I_2_ added had a high fill factor of 79.54%. Also the starch complex can form hydrogen bonds with the perovskite film to passivate surface defects. Additionally, the starch-I_2_ complex can absorb migrating I^−^ from perovskite to form a starch-I_3_^−^ complex. This complex can repress migration of ions in the films and then release the iodide ion to initiate the self-healing of iodide vacancies on the perovskite film. This resulted in the improved long-term stability of the resulting PSCs with the structure of glass/ITO/FAPbI_3_/Spiro-OMeTAD/Au. When exposed to 25 °C in 35% RH for 1500 h, the PSC with the addition of starch-I_2_ complex retained 91.12% of its initial PCE of 22.60%.

Likewise, the polymer additive dopants are also utilized for MAPbI_3_-based PSCs. Niu et al. incorporated polyvinylpyrrolidone (PVP) into the MAPbI_3_ precursor solution to enhance the crystallinity of the perovskite film [66]. With the addition of PVP to the perovskite precursor solution, the PVP can form an intermediate adduct and temporarily bonds with the MAI before crystallization to control the grain growth of perovskite. This results in enhanced grain sizes and fewer defects than the control. The treatment also gave the solar cells the ability to greatly withstand exposure to moisture in the air. PVP was found to form hydrogen bonds with the water molecules, preventing contact between the perovskite and the moisture. In other words, the PVP addition helps control crystal size, improve film uniformity, bolster resistance to humidity, and enhance stability.

For a CH_3_NH_3_PbI_3_ (MAPbI_3_) perovskite, moisture-induced degradation causes CH_3_NH_3_I and PbI_2_ to form. Because CH_3_NH_3_I is unstable, it degrades further into CH_3_NH_2_ and HI, which is irreversible in most conditions, as O_2_ and UV light react with HI. PVP can be added to the perovskite film to minimize the decomposition of MAPbI_3_ perovskite. The abundance of polar carbonyl functional groups forms hydrogen bonds with the NH_2_ of MA^+^ cations, which suppresses the decomposition of MAPbI_3_. PVP anchors MAI molecules through hydrogen bonding between the carbonyl groups of PVP and NH_2_ groups of MA^+^, which rearranges and recombines after moisture-induced degradation. The addition of PVP polymer lowered the formation energy of MAPbI_3_, resulting in the rapid recovery of the film in approximately 30 s when exposed to high moisture and subsequently removed from the humid condition. Additionally, after being sprayed with and exposed to water for 1 min, PSCs then self-healed at room temperature. After three cycles, PVP-treated PSCs restored up to 80% of their initial PCE, whereas the control cells completely degraded. The resulting device with the structure of glass/FTO/c-TiO_2_/PVP-MAPbI_3_/m-TiO_2_/Spiro-OMeTAD/Au had a PCE of 20.32% with improved operational and moisture stability. Specifically, the PVP-doped PSCs retained 90% of their initial PCE when exposed to 65% ± 5% RH for 500 h, which recovered rapidly after removal from the humid conditions.

**Figure 12:**
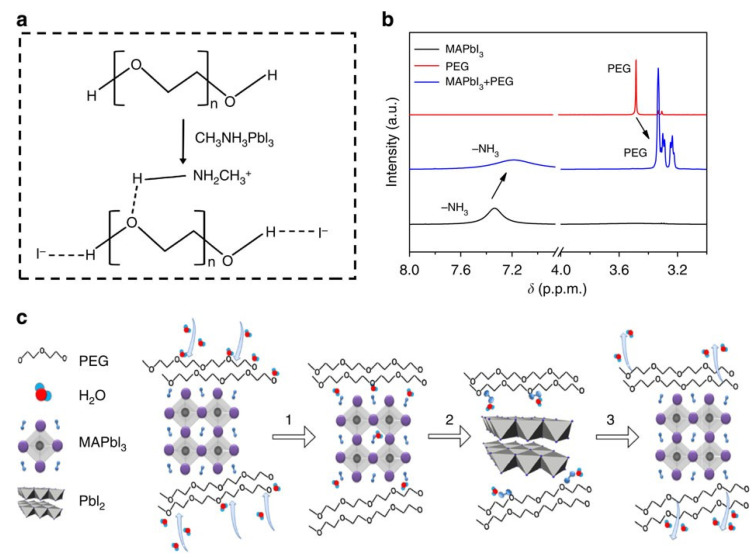
(**a**). Schematic illustration of hydrogen bond formation between PEG molecules and MAPbI_3_. (**b**). Comparison of ^1^H NMR spectra (3.0–8.0 ppm) for three samples: deuterated DMSO solutions containing MAPbI_3_ alone, a MAPbI_3_ and PEG mixture, and PEG alone. (**c**). Schematic depiction of the self-healing mechanism in PPSCs: (1) Water is absorbed onto the perovskite surface; (2) Hydrolysis of perovskite occurs, forming PbI_2_ and MAI·H_2_O; (3) PEG binds strongly with MAI, preventing its evaporation. Upon removal of the water vapor, the retained MAI reacts with nearby PbI_2_ to re-form perovskite in situ [67].

Zhao et al. conducted pioneer work and synthesized polyethylene glycol (PEG) as a self-healing polymer additive for MA-based PSCs [67]. The PEG monomers were added to the MAPbI_3_ precursor solution to improve the humidity stability of the resulting devices. When water-exposed perovskite degrades into PbI_2_ and MAI, the PEG polymer is able to form hydrogen bonds with the MA^+^ of the perovskite film to anchor onto the grains, which prevents MAI from dissolution, as shown in Figure 12. Once water evaporates and the film is no longer exposed to water, PEG-anchored MA^+^ reacts with PbI_2_ nearby, reversing the water-induced degradation. This results in self-healing from moisture-caused degradation. The resulting device with the structure of glass/FTO/TiO_2_/PEG-MAPbI_3_/Spiro-OMeTAD/Au had a PCE of 16% with improved moisture stability. When PEG-treated PSCs were exposed to 70% RH for 300 h, they maintained 65% of their original PCE; meanwhile, the control device lost most of its PCE within 50 h.

**Figure 13:**
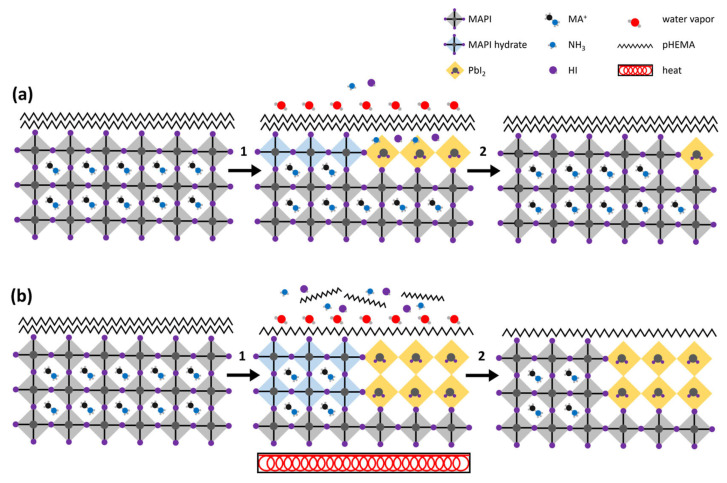
(**a**) The illustration displays the self-healing process of pHEMA-incorporated MAPI film when exposed to water. Step 1 represents the introduction of water, and Step 2 indicates the removal of water. (**b**) The schematic illustrates the process of pHEMA-added MAPI film as it self-heals after introduction and removal of heat [68].

Zhao et al. added poly(2-hydroxyethyl methacrylate) (pHEMA) to the MAPbI_3_ perovskite precursor solution to improve the quality of the perovskite film and induce self-healing property from moisture to the resulting PSCs [68]. The hydrophilic property of pHEMA means that it attracts and absorbs water, as shown in Figure 13. As a result, when MAPbI_3_ film is in a high-humidity environment, such as 70% RH, pHEMA absorbs moisture and forms hydrogen bonds with nitrogen and iodine degradation products that result from the moisture-induced breakdown of MAPbI_3_ film. Once water is evaporated from the film, these products are reintegrated into the perovskite structure, effectively self-healing the damaged film. To test the self-healing ability of pHEMA-treated PSCs, the films were exposed to 70% and 20% RH conditions alternatively for 28 days. According to the PbI_2_/MAPbI_3_ peak intensity ratio from X-ray diffraction, the films showed significant degradation at 70% RH but demonstrated self-healing at 20% RH as PbI_2_ converted back to MAPbI_3_.

**Figure 14:**
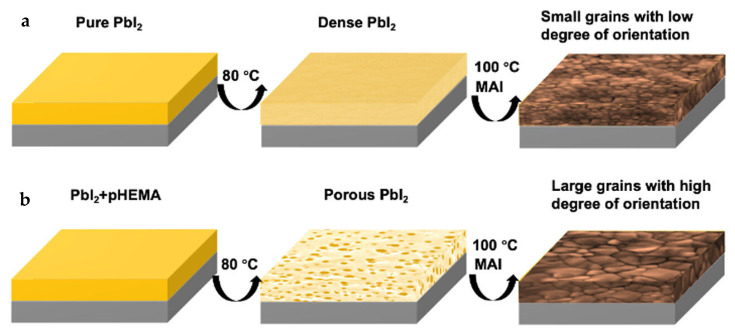
(**a**) The illustration displays the process of fabricating PbI_2_ film without pHEMA. The pure PbI_2_ film has small grains with a lower degree of orientation compared to its counterpart with the polymer additive. (**b**) The schematic illustrates the process of fabricating PbI_2_ film with pHEMA. The film with pHEMA added to the perovskite precursor solution has large grains with a higher degree of orientation than its pure counterpart [68].

Additionally, the doping of pHEMA into MAPbI_3_ leads to a porous PbI_2_ film, which results in pores that are more uniformly distributed on the surface of the PbI_2_ film. As shown in Figure 14, the grain size of the perovskite films is increased, which improves the fill factor and mobility of charged carriers, as trap densities and non-radiative recombination are lowered. The resulting device with the structure of glass/SnO_2_/pHEMA-MAPbI_3_/Spiro-OMeTAD/Ag had a PCE of 17.8% with improved humidity and thermal stabilities. For instance, pHEMA-treated PSCs retained 90.2% of their initial PCE when exposed at 70% RH for 500 h.

#### 3.1.2. Triple-Cation Perovskites

Unlike methods of addition of polymers to perovskite precursors mentioned in previous sections, polymer additives can be utilized as a template for an electron transport layer (ETL) or hole transport layer to improve the photovoltaic performance of the resulting devices. For example, Lalpour et al. utilized P123 triblock copolymer (CT) as a template for the TiO_2_ ETL to create a copolymer-templated TiO_2_ (CT-TiO_2_) [69]. Randomly porous TiO_2_ ETL has a high refractive index, which hinders the transmittance of light and decreases the amount of light reaching the active perovskite layer. This results in lower photocurrent density and efficiency. The usage of CT as a template for the TiO_2_ sol–gel solution improves the light transmittance, improving the efficiency of the resulting PSCs.

In addition to improving the photovoltaic performance of PSCs with CT-TiO_2_ ETL, the copolymer introduces self-healing properties to CT-treated PSCs. This copolymer contains multiple functional groups, such as hydroxyl, carbonyl, and amine, which form hydrogen bonds with the perovskite film. As a result, the non-covalent hydrogen bonding between the perovskite and CT-TiO_2_ anchors displaced ions of perovskite that are created when the film is damaged from stress. The anchored ions can reverse to their original state through the hydrogen bonding interaction, which leads to recrystallization and improved photovoltaic performance after exposure to stress conditions. The resulting device with the structure of glass/FTO/CT-TiO_2_/Cs_0.05_(MA_0.17_FA_0.83_)_0.95_Pb(I_0.83_Br_0.17_)_3_/Spiro-OMeTAD/Au demonstrated improved ambient stability. For instance, the unsealed CT-TiO_2_ PSC retained all of its initial PCE when exposed to 25–30 °C and RH of 30–45% for 90 days. The PCE increased from 11.08% to 11.27% over the duration of the stability testing due to the self-healing properties of CT copolymer.

Nevertheless, the addition of polymer monomers to the perovskite precursor to dope the perovskite film is still a common method researchers employ. For instance, Niu et al. incorporated acrylamide (AAm) monomers to Cs_0.05_(FA_0.90_MA_0.10_)_0.95_Pb(I_0.90_Br_0.10_)_3_ perovskite precursor solution to improve the humidity stability of resulting PSCs [70]. After thermal annealing of the precursor, perovskite-polymer hybrids form, where hydrogen bonds of multifunctional polymer with groups such as amide, carbonyl, etc., interact with surface defects such as iodine vacancies through Lewis acid–base interaction to passivate those defects and reduce trap densities. When the hybrid is exposed to water, polyamides in the hybrid form hydrogels prevent the dissolution of Pb^2+^ from perovskite into water, improving the moisture stability of the resulting device. When AAm-treated PSCs were immersed in water for 24 h, the rejection rate of Pb^2+^ dissolution was up to 94%. This is equivalent to the exposure of unprotected solar panels to rain for 24 h, which indicates that the AAm addition to the perovskite precursor solution is a viable method to improve the moisture stability of PSCs. The resulting device had a structure of ITO/NiOx/PTAA/Cs_0.05_(FA_0.90_MA_0.10_)_0.95_Pb(I_0.90_Br_0.10_)_3_/PCBM/BCP/Ag and demonstrated a PCE of 22.1%.

In short, rigid PSCs benefit from the addition of self-healing polymers. With introduction of hydrogen bonding, self-healing polymer improves the long-term operational stability and environmental stability, such as moisture resistance. Many researchers utilize polymers with functional groups, such as carboxyl, thiourea, etc., to establish hydrogen bonding capabilities with the perovskite structure. This leads to the dynamic reversal of damaged perovskite film under ambient conditions without external stimuli. Not only does this approach incorporate self-restorative properties to PSCs, but it also has the potential to passivate defects on film surface and at grain boundaries and prevent ion migration, further improving the overall photovoltaic performance of rigid PSCs.

### 3.2. Chemical Bonds

In the pursuit of enhancing the mechanical durability and environmental stability of rigid perovskite solar cells, the incorporation of self-healing polymers with chemically driven healing mechanisms has gained significant attention [71]. These polymers function through dynamic covalent bonds or reversible chemical interactions, particularly disulfide exchange [72], allowing the perovskite material to repair damage autonomously at the molecular level. When microcracks or mechanical stress compromise the perovskite layer or its interfaces, these chemical bonds can re-form under mild energy input, often heat or irradiation energy, which can restore structural integrity and device performance. In rigid PSCs, where mechanical flexibility is limited, such chemical self-healing mechanisms are especially valuable for mitigating long-term degradation caused by thermal cycling or environmental exposure. This approach not only improves PSC longevity and operational stability but also contributes to the development of more resilient and commercially viable perovskite-based photovoltaics.

Self-healing polymers with disulfide bonds are a promising method of improving the stability of rigid PSCs under operational or humid conditions. Polymer additives with disulfide bonds are capable of engaging in reversible exchange reactions through the formation of free radicals or nucleophilic thiol-disulfide substitution reactions [73]. When exposed to environmental degradation stimuli, interfacial defects form in the perovskite structure. In the presence of external restoring stimulus such as light or heat, thiolate ions or thiol radicals are formed, which then cleave existing disulfide bonds and undergo disulfide metathesis. This results in the polymer network being able to reorganize and reform broken linkages in order to repair degradation-induced damage.

When incorporated into PSCs, disulfide bonds repair structural damage and have even been found to be able to reverse defects formed during film creation. Additionally, disulfide bonds have the capability to hinder defects before they form. Many studies indicated that the addition of disulfide bond-containing polymers to PSCs mitigates degradation pathways, improving the photovoltaic performance of PSCs over operational time. As a result of these benefits, dynamic disulfide bond-based polymers have been a large focus for performance recovery of PSCs.

#### 3.2.1. Single-Cation Perovskites

Similar to other research groups that utilize polyurethane (PU) or its derivatives with disulfide bond to leverage their ability for self-healing PSCs, Zhang et al. tested doping a range of 0.05, 0.1, 0.15, 0.25, and 0.5 mg per mL solutions of PU to CsPbIBr_2_ perovskite precursor solutions [74]. By incorporating the PU into the perovskite, the group was able to observe reductions in defects, enabling the resulting PSC to recover 90% of its initial PCE. Additionally, it proved beneficial for lengthening the lifespan of PSCs under suboptimal heat and humidity conditions.

**Figure 15:**
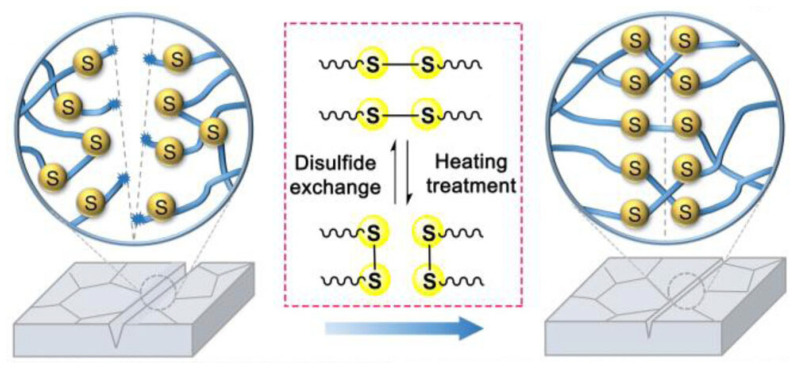
The diagram illustrates the heat-induced self-healing of PU-treated PSCs through the exchange of disulfide bonds [74].

Through Lewis acid–base interactions, carbonyl functional groups in PU and undercoordinated Pb^2+^ ions in the perovskite interact to repair defects in the perovskite film, which leads to greater structural integrity and improved efficiency. Additionally, disulfide groups in PU can break down under high-temperature conditions and reform to repair the device damage, which can be seen in Figure 15. With the addition of this polymer, PSCs were shown to have improved resistance to environmental factors, which lasted for over 35 days under 85 °C and over 80 days under 5% humidity at ambient atmosphere. The resulting device with the structure of glass/FTO/*c*-TiO_2_/PU-CsPbIBr_2_/Carbon had a PCE of 10.61%.

Likewise, Zheng et al. synthesized fullerene-derivatized polyurethane (C_60_-PU) with polyethylene glycol (PEG 2000), fullerene derivative C_60_-MPE-OH, hexamethylene diisocyanate, and 2-hydroxyethyl disulfide as precursors [75]. The urethane linkage in polyurethane is flexible and interacts with perovskite material through hydrogen bonding, which allows the polymer chains to rearrange and restore to the polymer matrix after damage, supporting self-healing of damaged perovskite film. When tested in contact with water, the perovskite film with C_60_-PU added was found to be more hydrophobic than the control device, which bolsters the device’s resistance to humid operating conditions.

One disadvantage of a traditional PU is that it has low mobility of electrons. As a result, the addition of fullerene (C_60_) mitigates the limitations of the usage of PU as a polymer additive for PSCs. The fullerene of the polymer has π-conjugated systems, which increase the efficiency of electron transport. Additionally, C_60_ has Lewis acid properties; it can chelate Pb^2+^ ions to reduce migration ions and stabilize grain boundaries.

Fr disulfide bonds self-heal during operational conditions; heat stimuli of 80 °C are needed in this study to initiate self-healing. When disulfide bonds are broken due to external conditions, they form thiol radicals, which can recombine at thermal stimuli to restore the polymer matrix. Broken disulfide bonds cross-link with adjacent chains, healing fractures on the film with the introduction of heat stimuli. The resulting device with the structure of glass/FTO/SnO_2_/C_60_-PU-FAPbI_3_/Spiro-OMeTAD/Ag had a PCE of 21.36% with enhanced ambient stability over 25 days.

#### 3.2.2. Double-Cation Perovskites

In a similar fashion, Wang et al. synthesized hindered urea/thiocarbamate bond Lewis acid–base material (HUBLA) to deposit on FAPbI_3_ perovskite film [76]. The polymer film was deposited on the perovskite absorbing layer. Under the right conditions, the HUBLA breaks down into its component bonds, then reintegrates and bonds with defects within the perovskite. Through the incorporation of hindered urea and thiocarbamate-based materials, whose disulfide bonds can break down and reform, HUBLA has been successfully demonstrated to repair damages in the perovskite structure. Under humid conditions, broken down NCO-AS reacts with water to reform into NH-AS that can fill vacancies in the perovskite structure. Additionally, HUBLA stressed by heat breaks down to form a compound that bonds with I_2_ within the perovskite material, which would otherwise lead to defects and aging of the material. Through these reactions, HUBLA can counteract the negative effects of many environmental conditions that typically accelerate the aging of perovskite.

With the addition of HUBLA, the treated device with the structure of glass/ITO/SnO_2_/FA_0.72_MA_0.28_PbI_3_/HUBLA/Spiro-OMeTAD/MoO_3_/Ag had a steady-state PCE of 24.5% that outperformed the control perovskite with a PCE of 21.7%. Additionally, over 3600 h of 25 °C and 30% RH conditions, HUBLA devices maintained 87% of their original efficiency, far outperforming the 43% drop in efficiency shown in the control. In 85 °C conditions, the HUBLA devices were shown to be capable of maintaining 90% of their original efficiency.

Kim et al. synthesized S-polymer for its dynamic disulfide bonds to incorporate moisture-induced self-healing properties to PSCs [77]. S-polymer is made up of cyclic dithiocarbonate monomer and oxathiolane-2-thione, and it is polymerized via living cationic ring-opening polymerization. This polymer is characterized by its dithiocarbonate groups, which contain thiocarbonyl functional groups. These functional groups enable moisture-dependent interaction with perovskite. Specifically, the backbone S-polymer contains disulfide bonds, which allow the polymer to absorb moisture at high humidity and chelate with undercoordinated lead ions and halides at the grain boundary. The chelation inhibits PbI_2_ formation, which is a byproduct of perovskite degradation due to moisture or heat. Once moisture is evaporated, the degradation byproducts are released and reintegrated into the perovskite lattice, reversing the breakdown and leading to reduced reactivity to water or oxygen to improve moisture and atmospheric stability.

In addition to facilitating the self-healing of perovskite film, S-polymer passivates halide vacancies, reducing trap densities and non-radiative recombination and forming hydrogen bonds with MA^+^ or FA^+^ ions to prevent the migration of ions and enhance the stability of the perovskite lattice. The addition of S-polymer enhanced the grain size and reduced defects at grain boundaries, which resulted in a higher short-circuit current (J_SC_) and fill factor (FF). However, relatively weak interaction between the perovskite film and the dithiocarbonate group of S-polymer resulted in a moderate enhancement of open-circuit voltage compared to the increase in J_SC_ and FF. The resulting PSCs with the structure of glass/FTO/*c*-TiO_2_/Li-TFSI-*mp*-TiO_2_/S-polymer-FA_0.65_MA_0.35_PbI_3−δ_Cl_δ_/n-OAI/Spiro-OMeTAD/Au had a PCE of 23.52%, which is significantly higher than the 19.70% PCE of the control device.

In conclusion, disulfide bond-based polymers can provide a variety of beneficial properties to rigid PSCs. By adding heat to these polymers, it is possible to regain most of the lost efficiency of a device over its entire operational lifespan, with some maintaining upwards of 90% of their initial PCE. Additionally, these polymers have the capacity to reform defects formed in the initial creation of the device, improving peak PCE alongside long-term PCE degradation. Due to their strength in these properties, polyurethane with disulfide bond and HUBLA have become a large focus of research in this area. Future research could also focus on developing a polymer with similarities to both of these materials, taking advantage of the customizability of the polyurethane family of materials and the compatibility of HUBLA’s component molecules with the defect species found in perovskites. Further research could also focus on discovering polymers that take advantage of both physical and chemical bonds, similar to those used in FPSCs, that are compatible with rigid PSCs. Table 2 provides a side-by-side comparison of the dominant degradation mechanisms in flexible versus rigid PSCs, along with the types of self-healing polymer chemistries typically employed for each system. This table highlights how mechanical bending and fatigue dominate in flexible PSCs, favoring elastic or hydrogen-bonding systems. Meanwhile, humidity and thermally induced degradation dominate in rigid PSCs, where more hydrophobic, crosslinked, or dynamic covalent systems are typically used.

It is worth mentioning that the current research studies utilize several quantitative approaches for evaluating self-healing efficiency, and these methods vary significantly. Common methods include mechanical recovery measured through bending-cycle endurance or AFM/SEM crack analysis, recovery ratio of photovoltaic performance (PCE, VOC, JSC, and FF), and defect-passivation or charge-transport recovery. However, because each study uses different testing conditions, such as strain rate, humidity level, illumination intensity, and bending radius, direct comparison of self-healing efficiency is difficult. As a result, standardized, quantitative measurements (such as %PCE retention after N bending cycles at a defined strain) for reliably evaluating healing efficiency and long-term performance retention should be established for a more reliable comparison.

## 4. Conclusions and Outlook

Perovskite solar cells have significantly improved in efficiency and stability in recent years, bringing them closer to commercial viability and expanding their potential applications across a range of optoelectronic technologies. However, mechanical fragility and sensitivity to environmental conditions continue to pose challenges to their long-term operational durability. In response, self-healing polymers have emerged as a promising solution for enhancing the mechanical and environmental resilience of flexible and rigid PSCs, especially in the development of flexible and wearable solar cell technologies, where stress and strain are frequent.

By harnessing dynamic reversible bonding mechanisms, such as covalent disulfide exchange and non-covalent hydrogen bonding, researchers have explored the potential of these polymers to promote autonomous repair of damage in both rigid and flexible PSCs. These self-healing functionalities are primarily utilized in flexible PSCs to alleviate mechanical stress and prevent crack propagation in the perovskite film during bending or stretching. In rigid PSCs, on the other hand, they serve to mitigate moisture-induced degradation, a major cause of performance loss under real-world operating conditions.

This review identified two primary categories of self-healing polymer additives: those utilizing dynamic covalent disulfide bonds and those incorporating reversible hydrogen bonding networks. Each system demonstrates distinct mechanisms, advantages, and limitations. For example, disulfide-based systems often offer stronger chemical recovery under thermal stimuli, while hydrogen bond-based polymers excel in low-energy, room-temperature healing. This review categorized these polymers first by the substrate type (rigid or flexible), then by the type of self-healing bonding mechanism utilized, and finally by the perovskite composition used in the absorber layer for clear context organization.

According to the findings of this review, the incorporation of self-healing polymers is a viable and increasingly practical technique to improve the long-term stability and performance of PSCs. In order to design effective self-healing systems, a deep understanding of how healing mechanisms interact with degradation pathways in PSCs is essential. Researchers continue to engineer these materials to self-sufficiently repair mechanical damage, interfacial defects, and microcracks that frequently arise due to environmental exposure and repeated stress cycles. While these challenges are more pronounced in flexible devices, they are still of significant concern in rigid PSCs operating under variable environmental conditions.

Ideally, these polymers should integrate restorative functionality without compromising device efficiency. However, in practice, incompatibilities between the polymer and the active or charge transport layers may result in decreased charge mobility, phase separation, or additional degradation pathways. Specifically, the influence of polymer additives on compatibility with transport layers is highly dependent on the device structure and materials chosen for the specific PSC. Different polymers interact with the perovskite lattice in distinct ways, which largely depend on whether the polymer coordinates with undercoordinated ions, such as Pb^2+^ or halide vacancies, within the perovskite film. When the coordination is favorable, the incorporation of the polymer can reduce trap densities, decrease non-radiative recombination, and therefore improve the carrier extraction at the transport-layer interfaces. Consequently, it is critical to select or design polymer additives that are chemically and physically compatible with the device stack. In rigid PSCs, appropriate polymer systems have been shown to improve stability under humid conditions, while, in flexible PSCs, they alleviate fatigue and enhance mechanical reliability under stress.

Across the studies examined, several overarching design guidelines emerge for synthesizing and integrating self-healing polymers into PSCs. First, the dynamic bonding mechanism should be matched to the main degradation method of the device: fast-exchanging hydrogen bonds benefit FPSCs with bending-induced microcracks, while dynamic covalent bonds positively impact rigid PSCs requiring long-term stability under thermal stress or humidity. Then, the compatibility between the polymer and perovskite should be maximized when the polymer does not disrupt the crystallization of perovskite, and even positively impacts the charge transport. Self-healing polymers with both non-covalent physical bonds and covalent chemical bonds are most efficient for PSC performance recovery since they require less initial energy and maintain a higher PCE over long durations of time as compared to single-bond type counterparts. Because of increased complexity of molecular structure, these polymers have to be curated for compatibility with other compounds in PSCs. Together, these insights provide predictive guidelines for synthesizing the next-generation self-healing additives tailored to the substrate type, perovskite composition, and device architecture.

Furthermore, multifunctional self-healing polymers show promise in addressing multiple performance bottlenecks simultaneously. For instance, in addition to mechanical healing, certain polymers enhance encapsulation or passivate grain boundaries and surface defects in the perovskite layer, contributing to improved charge extraction and overall photovoltaic performance. Based on the data and case studies analyzed in this review, self-healing polymer additives can be adapted to a wide variety of perovskite compositions and device architectures. Additionally, these self-healing polymers demonstrate strong potential for broader applications across other perovskite-based optoelectronic devices, such as light-emitting diodes, laser diodes, optical sensors, optical modulators, and stretchable photodetectors. As the field of perovskite-based photovoltaics moves toward more durable, scalable, and multifunctional device platforms, the integration of self-healing polymers represents a more comprehensive pathway to stabilizing PSCs without compromising their optoelectronic performance.

## Figures and Tables

**Table 1:** Comparison of characteristic timescales and energetic requirements for hydrogen bonding and disulfide exchange in self-healing polymer systems.

Healing Mechanism	Characteristic Timescale	Energetic Requirement	Primary Function
Hydrogen bonding	Seconds to minutes (fast)	Low activation energy	Rapid crack closure; immediate mechanical recovery
Disulfide exchange	Minutes to hours (slow)	Moderate to high activation energy	Long-term polymer network rearrangement; durable recovery

**Table 2:** Comparative summary of main degradation modes and preferred polymer chemistries for flexible and rigid perovskite solar cells.

Device Type	Main Degradation Modes	Preferred Polymer Chemistries
Flexible PSCs	Mechanical bending fatigue, crack initiation/propagation, interfacial delamination	Elastomeric polymers, hydrogen-bonding systems (fast recovery), dynamic covalent networks with high elasticity, PU-based systems
Rigid PSCs	Moisture-induced degradation, phase instability, grain-boundary expansion, and mechanical microcracks from thermal cycling	Hydrophobic polymers, disulfide bond systems (long-term healing), crosslinked networks, passivation-oriented polymers (amine/carbonyl coordination)

## Data Availability

No new data were created or analyzed in this study.
